# A lateral signalling pathway coordinates shape volatility during cell migration

**DOI:** 10.1038/ncomms11714

**Published:** 2016-05-26

**Authors:** Liang Zhang, Valbona Luga, Sarah K. Armitage, Martin Musiol, Amy Won, Christopher M. Yip, Sergey V. Plotnikov, Jeffrey L. Wrana

**Affiliations:** 1 Center for Systems Biology, Lunenfeld-Tanenbaum Research Institute, Mount Sinai Hospital, Toronto, Ontario, Canada M5G 1X5; 2 Department of Molecular Genetics, University of Toronto, Toronto, Ontario, Canada M5S 1A8; 3 Department of Cell & Systems Biology, University of Toronto, Toronto, Ontario, Canada M5S 3G5; 4 Institute of Biomaterials and Biomedical Engineering, The Terrence Donnelly Centre for Cellular and Biomolecular Research, Department of Biochemistry, University of Toronto, Toronto, Ontario, Canada M5S 3E1

## Abstract

Cell migration is fundamental for both physiological and pathological processes. Migrating cells usually display high dynamics in morphology, which is orchestrated by an integrative array of signalling pathways. Here we identify a novel pathway, we term lateral signalling, comprised of the planar cell polarity (PCP) protein Pk1 and the RhoGAPs, Arhgap21/23. We show that the Pk1–Arhgap21/23 complex inhibits RhoA, is localized on the non-protrusive lateral membrane cortex and its disruption leads to the disorganization of the actomyosin network and altered focal adhesion dynamics. Pk1-mediated lateral signalling confines protrusive activity and is regulated by Smurf2, an E3 ubiquitin ligase in the PCP pathway. Furthermore, we demonstrate that dynamic interplay between lateral and protrusive signalling generates cyclical fluctuations in cell shape that we quantify here as shape volatility, which strongly correlates with migration speed. These studies uncover a previously unrecognized lateral signalling pathway that coordinates shape volatility during productive cell migration.

## Abstract (web summary)

Migrating cells display dynamic morphologies that are coordinated by signalling pathways. Here the authors identify a lateral signalling pathway, comprised of the planar cell polarity protein Pk1 and Arhgap21/23, that regulates fluctuations in cell shape during productive cell migration.

## Graphical abstract

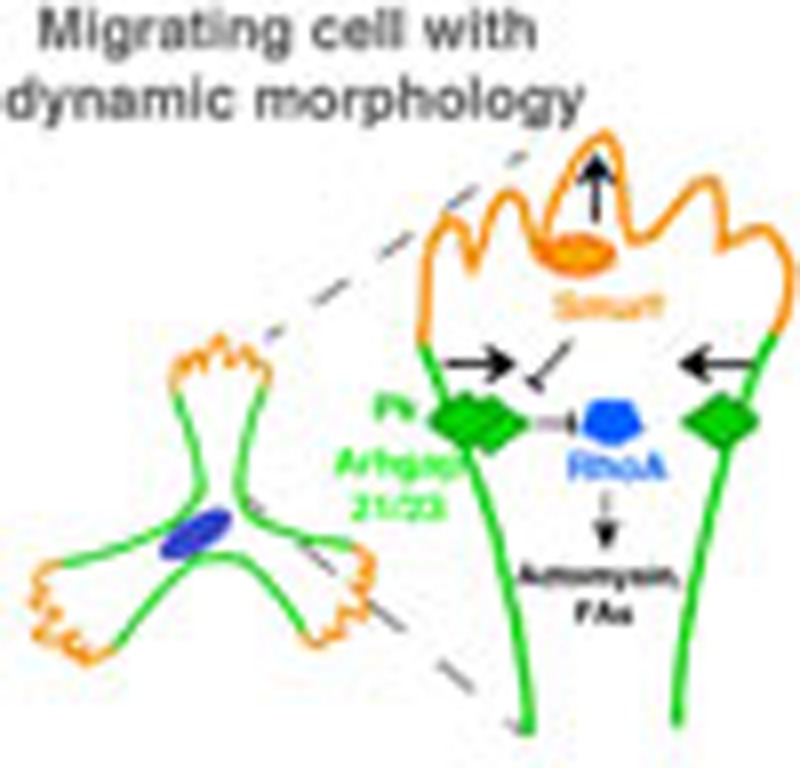

Cell migration plays an essential role in embryonic development and physiological homeostasis and underlies pathological mechanisms in many diseases, including cancer metastasis^1^. Migrating cells often display dynamic morphologies that encompass formation of protrusions and adhesions at the leading front in conjunction with disassembly of adhesions and body retraction at the rear. In general, this has been termed front–rear polarity^2^. Studies have identified a plethora of signalling mechanisms that regulate the dynamic asymmetry of cellular structures and activities along the front–rear axis during migration. Intriguingly, many signalling networks that orchestrate asymmetry in migrating cells are also essential for establishing epithelial apical–basal polarity^2^,^3^,^4^,^5^.

Planar cell polarity (PCP) refers to the asymmetric distribution of cellular activities and structures within the epithelial plane that is orthogonal to the apical–basal axis. PCP signalling is essential for tissue morphogenesis during development and depends on a conserved group of core proteins including transmembrane proteins Frizzled (Fzd) and Van Gogh-like (Vangl), as well as cytoplasmic proteins Disheveled (Dvl), Diego and Prickle (Pk)^6^,^7^,^8^. These core PCP components are typically organized into asymmetric complexes along the tissue plane and impaired asymmetry causes disruption of planar polarity. Studies of PCP signalling also point to its important role in modulating cell migration^9^,^10^. For example, the convergent extension movement of mesodermal and neuro-ectodermal cells in vertebrates depends on proper PCP signalling^10^. Furthermore, recent studies identified various PCP components associated with cancer progression and indicate that PCP signalling is essential for cancer metastasis^11^,^12^,^13^. Importantly, asymmetric PCP complexes have been demonstrated in motile breast cancer cells (BCCs)^9^. However, the mechanisms that underlie PCP activity in cell migration are still unclear.

Here we report a novel pathway in migrating cells we term lateral signalling, which consists of Prickle1 (Pk1), a core PCP component, and Arhgap21/23, members of the GTPase-activating protein (GAP) family. In migrating cells, we find that Pk1 and Arhgap21/23 are located at non-protrusive membranes that are lateral to active protrusions. We show that Pk1–Arhgap21/23 function to confine protrusive activity through regulating RhoA and thus organization of the actomyosin network, focal adhesion (FA) dynamics and mechanical properties of cell membrane. We further demonstrate that lateral signalling is required for fluctuations in cell morphology during migration and quantify these dynamic changes as shape volatility, a parameter that measures fluctuations in the aspect ratio (AR) of cells during migration. Shape volatility correlates strongly with cell migration and at the mechanistic level, is coordinated by the antagonistic interplay between non-protrusive lateral signalling by Pk1–Arhgap21/23 and protrusive signalling via Smurf2, an E3 ubiqutin ligase in the PCP pathway. Interestingly, while critical for cell migration speed, this lateral-protrusive asymmetry does not have an essential role in the directionality of cell migration during chemotaxis. These studies thus identify a novel lateral signalling system that coordinates shape volatility and functions orthogonally to the conventional front–rear polarity in driving productive cell migration.

## Results

### Pk1 mediates lateral signalling in migrating cells

**Figure 1:**
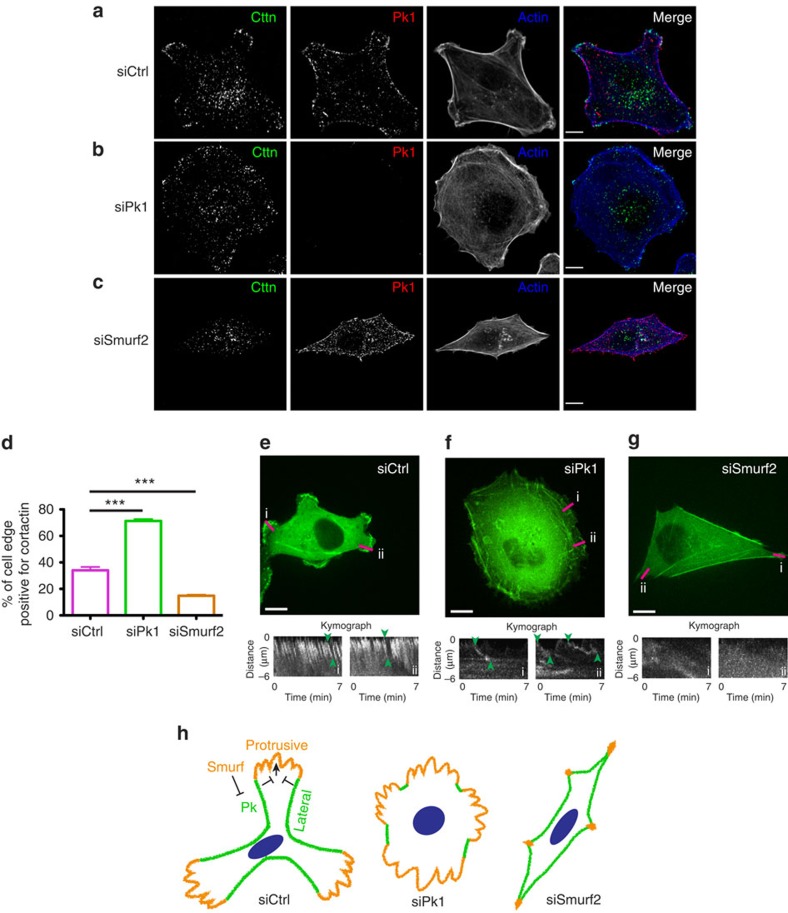
Pk1 mediates lateral signalling in migrating cells. (**a**–**c**) MDA-MB-231 cells were transfected with the indicated siRNAs. After 72 h, the cells were treated with ACM for 18 h. Confocal images of immunostained endogenous cortactin (Cttn; white, first column; and green, fourth column) and Pk1 (white, second column; and red, fourth column) are shown for the indicated conditions. The actin network was detected by phalloidin staining (white, third column; and blue, fourth column). (**d**) Quantification of the proportion of cell edge positive for cortactin signal (results represent the mean+s.e.m. of three independent experiments, *n*=30 cells for each condition in every experiment, ***P<0.001 with two-tailed unpaired *t*-test). (**e**–**g**) MDA/YFP-actin cells were transfected with indicated siRNAs. After 72 h, cells were treated with ACM and examined by time-lapse confocal microscopy. Representative snapshots are shown here, and kymograph analyses of the regions highlighted by the pink bars are shown below. Pairs of arrowheads highlight representative cycling periods of actin networks. Scale bar, 10 μm. (**h**) Schematic of Pk1-mediated lateral signalling that coordinates protrusive activity. Inhibition (for example, siPk1) or enhancement (for example, siSmurf2) of lateral signalling disrupts the balance between protrusive activity and non-protrusive lateral cortex.

The PCP pathway is essential for fibroblast exosome-driven BCC migration^9^. To investigate the mechanism, we examined the localization of Pk1, a core PCP component, in MDA-MB-231 BCCs stimulated with exosomes contained within active conditioned media (ACM) derived from fibroblast L cells^9^. On ACM treatment, BCCs typically display random migration, with multiple protrusions oriented in various directions (Fig. 1a and Supplementary Movie 1). Similar to previous studies^9^, Pk1 is detected on lateral cortex that flank the protrusive lamellipodia marked by cortactin, a regulator of the Arp2/3 complex^14^ (Fig. 1a). To investigate whether the lateral localization of Pk1 could reflect its signalling activity, we examined MDA-MB-231 cells after silencing Pk1 or Smurf2, which is an E3 ubiquitin ligase that targets Pk1 for degradation during PCP signalling^15^. Interestingly, Pk1-deficient cells displayed protrusive membranes that incorporate the majority of the cell periphery and was highlighted by expanded distribution of cortactin (Fig. 1b,d). In line with Smurf2-mediated Pk1 degradation^15^, Smurf2-deficient cells displayed enhanced Pk1 staining that extended into the polarized ends of the cell, along with intensified actin bundles (Fig. 1c). Importantly, Smurf2-deficient cells displayed significantly reduced peripheral cortactin, indicating inhibition of protrusive activity (Fig. 1c,d). Together these results suggest that laterally localized Pk1 and its regulation by Smurf2 play a vital role in coordinating protrusive activity in migrating cells. Importantly, silencing of Pk1 and Smurf2 both inhibited ACM-stimulated migration (Supplementary Fig. 1a–c), which are consistent with prior studies^9^, and suggest that coordinating protrusive activity is essential for cell migration.

To further study how Pk1 and Smurf2 regulate protrusive activity and cell migration, we examined actin network dynamics in MDA-MB-231 cells that stably express YFP-actin (MDA/YFP-actin). In response to ACM treatment, control MDA/YFP-actin cells typically generate multiple active protrusions. Time-lapse microscopy and kymograph analysis revealed that each protrusion contained condensed actin networks and displayed fast and periodic protrusion–retraction cycles (Fig. 1e and Supplementary Movie 1). Furthermore, individual protrusions were flanked by lateral cortex that was smooth and displayed little or no dynamics (Fig. 1e and Supplementary Movie 1). Strikingly, when Pk1 was knocked down, cells displayed protrusive membrane ruffling that expanded around the entire cell periphery (Fig. 1f and Supplementary Movie 2) and kymography revealed that the actin networks were less condensed than controls and the protrusion–retraction cycles were disrupted or irregular (Fig. 1f and Supplementary Movie 2). In contrast, knockdown of Smurf2 led to a remarkable opposite phenotype, as cells displayed static morphologies and no protrusive activity (Fig. 1g and Supplementary Movie 3).

Our results suggest that cell migration depends on a novel Pk1-mediated lateral signalling system that constrains protrusive, cortactin-positive lamellipodia (Fig. 1h). Lateral signalling in turn is negatively regulated by Smurf, which promotes cell protrusions^16^ (Fig. 1h). In this context, when protrusive signalling is inhibited (for example, on Smurf2 inhibition), lateral signalling predominates and the cells display little protrusive activity. In contrast, when lateral signalling is inhibited (for example, Pk1 loss), protrusive signalling predominates and the cells display expanded, albeit non-productive protrusive activity. Furthermore, these results indicate that the balance between protrusive-lateral signalling is critical for cell migration, since interference with either pathway inhibits migratory speed. To confirm that lateral signalling was manifested in other cells, we examined Pk1 localization in ACM-stimulated human SUM-159PT and mouse EMT6 cells, which showed Pk1 localization along the non-protrusive lateral cortex, similar to MDA-MB-231 cells (Supplementary Fig. 1d,e).

### A Pk–Arhgap21/23 complex regulates lateral signalling

**Figure 2:**
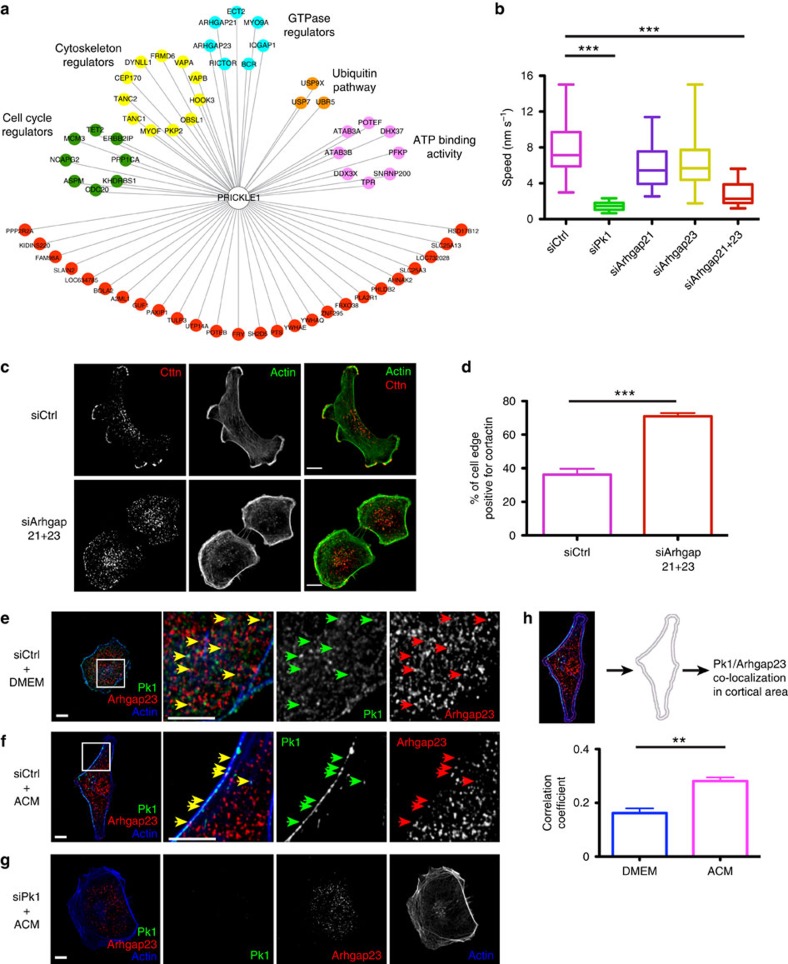
Pk1 interacts with Arhgap21/23 to regulate lateral signalling. (**a**) Pk1 interactome in MDA-MB-231 cells. Flag-immunoprecipitation was performed using MDA-MB-231 cells that stably express Flag-Pk1. Pk1-interacting proteins were identified using liquid chromatography–mass spectrometry (LC–MS) and annotated using the Functional Annotation Clustering tool in DAVID bioinformatics resources. Colour of nodes: green, cell cycle regulators; yellow, cytoskeleton regulators; blue, Rho GTPase regulators; orange, ubiquitin/proteasome regulators; pink, ATP-binding proteins; and red, other proteins. (**b**) MDA-MB-231 cells were transfected with the indicated siRNA to Pk1, Arhgap21, Arhgap23 or a control sequence (Ctrl). After 72 h, cells were treated with ACM for 18 h and cell migration speed was analysed (*n*=20 cells per group. ***P<0.0001 with two-tailed unpaired *t*-test. Results of one representative experiment from four biological repeats are shown). (**c**) MDA-MB-231 cells were transfected with the indicated siRNAs and treated with ACM as in Fig. 1a–c. Confocal images of immunostained endogenous cortactin (Cttn; red, first column; and white, third column) and phalloidin staining of actin (green, first column; and white, second column) are shown. Scale bar, 10 μm. (**d**) Quantification of the proportion of cell edge positive for cortactin signal (results represent the mean+s.e.m. of three independent experiments, *n*=30 cells for each condition in every experiment, ***P<0.001 with two-tailed unpaired *t*-test). (**e**–**h**) Confocal images of endogenous Pk1 and Arhgap23 are shown for MDA-MB-231 cells that were transfected with the indicated siRNAs and treated with control media (DMEM) or ACM. The co-localizations of Pk1 (green arrows) with Arhgap23 (red arrows) in the cortical region are indicated (yellow arrows) and quantified in **h** (results represent the mean+s.e.m. of four independent experiments, *n*=10 cells for each condition in every experiment. **P<0.005 with two-tailed unpaired *t* test). Note in cells with Pk1 silencing (**g**), Arhgap23 is depleted from the protrusive peripheral cortex. Scale bar, 10 μm.

To understand the mechanisms that underlie Pk1-mediated lateral signalling, we mapped Pk1 protein partners by affinity purification and mass spectrometry. Flag-tagged Pk1, stably expressed in MDA-MB-231 cells was immunoprecipitated and associated proteins were identified by mass spectrometry. Out of 63 Pk1-interacting proteins (Fig. 2a), we chose to focus on Arhgap21 and Arhgap23, which are little studied close homologues of the family of Rho GTPase-activating proteins (RhoGAPs)^17^. Individual knockdown of Arhgap21 or Arhgap23 had no significant effect on ACM-stimulated migration (Fig. 2b). However, Arhgap23 expression was enhanced following Arhgap21 knockdown (Supplementary Fig. 2a–c) and silencing both Arhgaps significantly inhibited migration of MDA-MB-231 cells (Fig. 2b), indicating redundancy. Importantly, similar to Pk1 deficiency, silencing of Arhgap21/23 led to expansion of protrusive activity around cell periphery, as indicated by cortactin staining (Fig. 2c,d). Furthermore, in MDA/YFP-actin cells, knockdown of Arhgap21/23 induced the characteristic round morphology and diffuse protrusive activity similar to Pk1 knockdown cells (Supplementary Fig. 2d and Supplementary Movie 4).

We next examined Arhgap23 localization in MDA-MB-231 cells, which showed prominent cortical and cytoplasmic staining that was lost following Arhgap23 knockdown (Supplementary Fig. 2e). Of note, the nuclear signal persisted in Arhgap23 knockdown cells, suggesting this reflects nonspecific staining (Supplementary Fig. 2e). In unstimulated MDA-MB-231 cells, we observed that Arhgap23 co-localized with Pk1 was primarily cytoplasmic (Fig. 2e), but on ACM treatment, was increased along the non-protrusive cortical region of migrating cells (Fig. 2f,h). Importantly, we did not observe significant changes in overall Pk1–Arhgap23 interaction by immunoprecipitation and immunoblotting (Supplementary Fig. 2g). Moreover, on Pk1 knockdown, Arhgap23 was absent from the periphery following ACM treatment (Fig. 2g), whereas Pk1 was still localized at the peripheral cortex when siArhgap21/23 were knocked down (Supplementary Fig. 2f). Together these results suggest that Pk1 recruits Arhgap23 to the lateral cortex to regulate cell migration.

**Figure 3:**
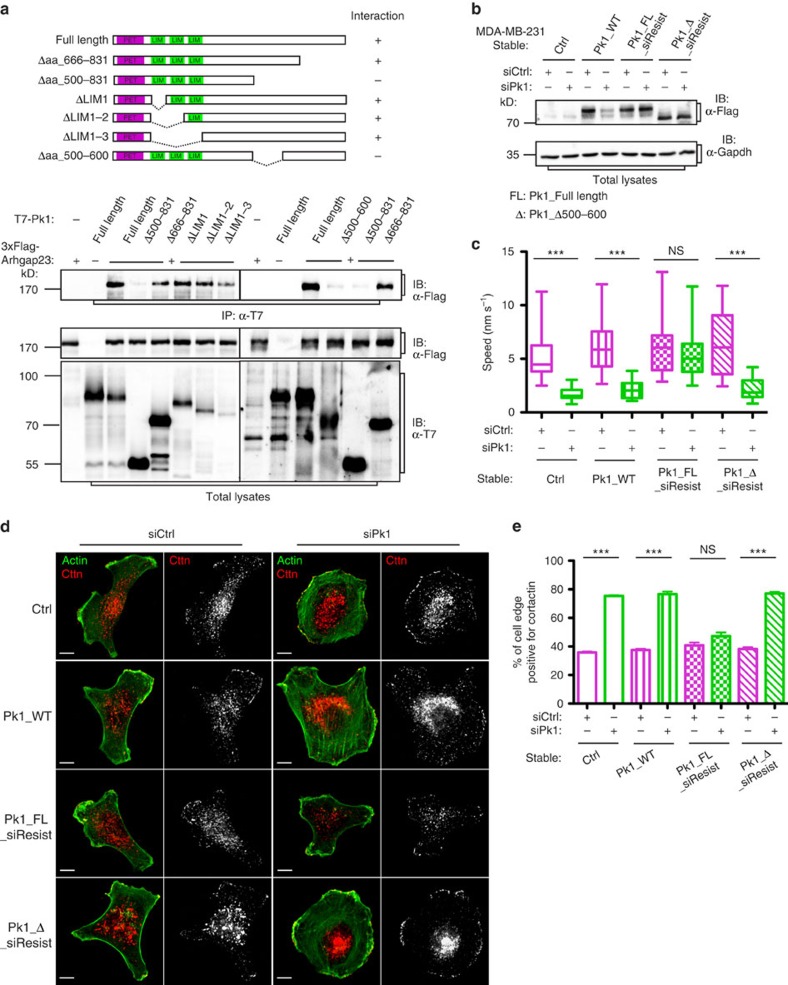
Pk1–Arhgap21/23 interaction is necessary for lateral polarity that regulates cell migration. (**a**) The region of Pk1 spanning amino acids 500–600 is required for mediating Pk1–Arhgap23 interaction. Cell lysates of MDA-MB-231 cells transfected with 3 × Flag-tagged Arhgap23 and T7-tagged Pk1 full-length or truncated constructs, as indicated in the top schematic, were subjected to anti-T7 immunoprecipitation (IP) and anti-Flag immunoblotting (IB). (**b**) Generation of MDA-MB-231 cells that stably express siRNA-resistant Pk1 constructs. MDA-MB-231 cells stably expressing Flag-tagged, full-length, wild-type Pk1 (Pk1_WT), full-length (Pk1_FL_siResist) or Δaa500–600 Pk1 (Pk1_Δ_siResist) mutants that are resistant to siRNA-mediated silencing, or control vector (Ctrl) were transfected with control siRNA (siCtrl) or siRNA that targets endogenous Pk1 (siPk1). After 72 h, cells were lysed and cell lysates were subjected to anti-Flag IB. (**c**) Pk1-Arhgap23 interaction is important for cell migration. Pk1_WT, Pk1_FL_siResist, Pk1_Δ_siResist or Ctrl MDA-MB-231 cells were transfected with control siRNA (siCtrl) or siRNA that targets endogenous Pk1 (siPk1). After 72 h, cell migration speed was analysed and plotted. *n*=20 cells per group. ***P<0.0001 with two-tailed unpaired *t*-test. Results of one representative experiment from four biological repeats are shown. NS (*P*=0.38) indicates not statistically significant with two-tailed unpaired *t*-test). (**d**,**e**) Pk1–Arhgap23 interaction is important for coordinating protrusive activity. Immunostaining of cortactin (Cttn) and phalloidin labelling of actin were performed on cells in the indicated conditions (**d**) and the proportions of cell edge positive for cortactin signal were quantified (**e**) (results represent the mean+s.e.m. of three independent experiments, *n*=30 cells for each condition in every experiment, ***P<0.0001 and NS (*P*=0.116) indicates not statistically significant with two-tailed unpaired *t*-test). Scale bar, 10 μm.

We next mapped the Pk1–Arhgap23 interaction using various deletion mutants of Pk1 (Fig. 3a). This showed that the middle region of Pk1 (amino acids 500–600; Pk1_Δ) mediates interaction with Arhgap23. To test whether the Arhgap23-binding mutant of Pk1 could support cell migration, we generated pools of MDA-MB-231 cells stably expressing siRNA-resistant Flag-tagged wild-type Pk1, or the Arhgap23-binding mutant (Pk1_FL_siResist or Pk1_Δ_siResist, respectively), as well as Flag-tagged siRNA-sensitive Pk1 (Pk1_WT), or empty vector (Ctrl), as controls (Fig. 3b). As expected, expression of siResist Pk1 rescued migration and cortactin distribution in siPk1-treated cells (Fig. 3c–e). In contrast, expression of the Arhgap23-binding mutant (Pk1_Δ) did not (Fig. 3c–e). Altogether, these data demonstrate that Pk1-dependent lateral signalling is dependent on its interaction with Arhgap23.

### Pk1–Arhgap signalling regulates RhoA

**Figure 4:**
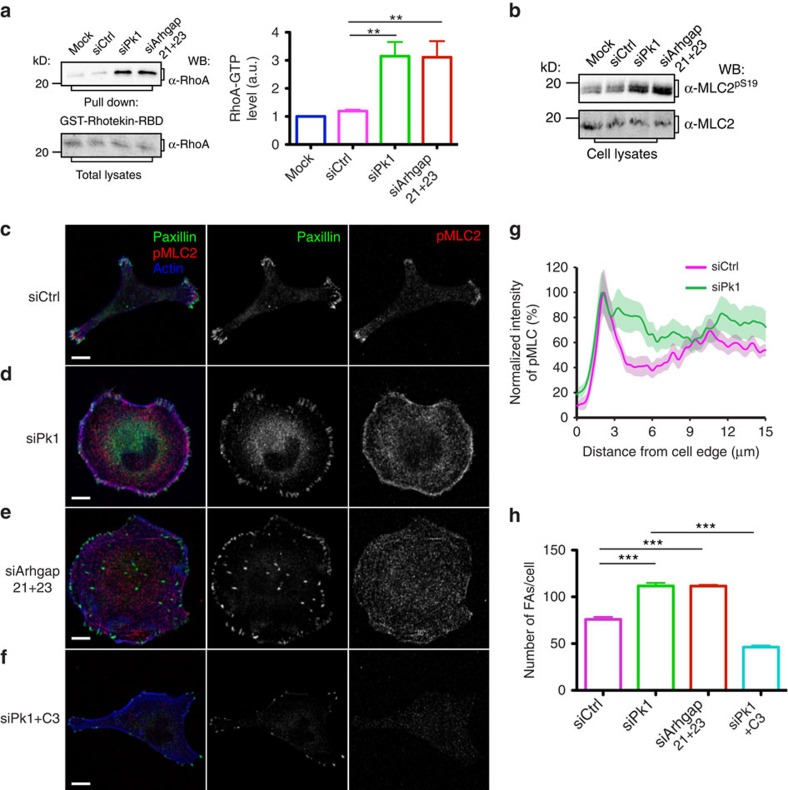
Lateral signalling regulates RhoA activity. (**a**) Active RhoA is enhanced after silencing of Pk1 and Arhgap21/23. MDA-MB-231 cells were transfected with the indicated siRNAs. Cell lysates were subjected to a pull-down assay using glutathione S-transferase (GST)-fused rho-binding domain (RBD) of Rhotekin that specifically interacts with GTP-bound RhoA. The precipitates were subjected to anti-RhoA immunoblotting (IB). Intensity quantifications are normalized to total RhoA and shown in the right panel (results represent mean+s.d., *n*=4 biological repeats, **P<0.005 with two-tailed unpaired *t*-test). (**b**) Silencing of Pk1 leads to an increased level of active MLC2. MDA-MB-231 cells were either mock transfected or were transfected with the indicated siRNAs. Cell lysates were subjected to an IB assay to analyse the level of active MLC2 with phosphorylation at serine 19. (**c**–**h**) Silencing of Pk1 and Arhgap21/23 disrupts the distribution of active MLC2 and FAs. Confocal images are shown for ACM-treated MDA-MB-231 cells that were transfected with the indicated siRNAs. Cells with Pk1 suppression were also treated with C3 transferase that inhibits the activity of RhoA. Active MLC2 (red, first column; and white, third column) was immunolabelled in parallel with endogenous paxillin (green, first column; and white second column). Counterstaining with phalloidin (blue, first column) was used to show the organization of the actin network. (**g**) Distribution of active MLC2 within 15 μm distance from the cell edge (results of one representative experiment from three biological repeats with similar results are shown, *n*=10 cells, lines represent mean and shaded area represents s.e.m.) and (**h**) the numbers of FAs/cell (results represent the mean+s.e.m. of four independent experiments, *n*=10 cells in each repeat, ***P<0.001 with two-tailed unpaired *t*-test) were quantified. Scale bar, 10 μm.

The Rho family of small GTPase cycle between an active GTP-bound form and an inactive GDP-bound form and play a key role in regulating cell morphology, migration and polarity^2^,^18^,^19^. The spatiotemporal asymmetry of RhoGTPase activity drives cell migration and is in large part regulated by GTP exchange factors and GAPs^20^,^21^,^22^. The interaction between Pk1 and Arhgap21/23 indicates a key role for RhoGTPases in lateral signalling. We therefore examined the activity of RhoGTPases in MDA-MB-231 cells. Following siRNA-mediated knockdown of either Pk1 or Arhgap21/23, the level of GTP-bound, active RhoA was significantly enhanced (Fig. 4a), and active Rac1 was either unchanged or decreased, respectively (Supplementary Fig. 3a). Similarly, MDA-MB-231 cells stably transfected with shRNA targeting Pk1 (MDA/shPk1) also demonstrated elevated active RhoA compared with controls (Supplementary Fig. 3b,c). Moreover, overexpressing Arhgap23 in MDA/shPk1 cells suppressed the elevation of active RhoA to a similar level as treatment with the active RhoA inhibitor, C3 transferase (Supplementary Fig. 3d). We also confirmed that the interaction between Pk1 and Arhgaps was important for Pk1-dependent regulation of RhoA (Supplementary Fig. 3e). Overall, these results show that lateral Pk1–Arhgap21/23 signalling restricts active RhoA.

### Lateral signalling regulates actomyosin and FAs

The assembly and contraction of the actomysin network and FA dynamics are regulated by RhoA and are key elements governing cell migration^23^. We therefore examined myosin light chain 2 (MLC2), which promotes actomyosin contractility, and is activated downstream of RhoA via phosphorylation on serine 19 by the Rho kinase, ROCK. In comparison to control cells, Pk1 and Arhgap21/23 knockdown cells displayed increased MLC2^pS19^ (Fig. 4b–e), consistent with elevated active RhoA in these cells. Localization of MLC2^pS19^ further revealed that in migrating control cells MLC2^pS19^ was concentrated within a narrow region in the lamella of protrusions (Fig. 4c,g), consistent with the role of active myosin in generating contractile lamella^23^. In contrast, MLC2^pS19^ was diffusely distributed around Pk1-deficient cells (Fig. 4d,g). Thus, loss of lateral signalling leads to expansion of activated RhoA and MLC2^pS19^ in Pk1-deficient cells.

During migration, Rho-mediated activation of actomyosin has an important role in generating the mechanical force that promotes the maturation of FAs^23^,^24^. Immunolabelling of paxillin, a structural component of FA, showed various-sized FAs in the protrusions of control cell (Fig. 4c), whereas in Pk1-deficient cells, increased numbers of FAs were observed around the cell periphery (Fig. 4d,h). Moreover, inhibition of RhoA using C3 transferase significantly decreased both FAs and MLC2^pS19^ levels in Pk1 knockdown cells (Fig. 4f,h). We also observed similar phenotypes of active MLC2 and FAs in cells with combinatorial knockdown of Arhgap21 and Arhgap23 (Fig. 4e). Together these data indicate that Pk1–Arhgap21/23 lateral signalling plays an important role in confining RhoA activity and regulating actomyosin and FAs in migrating cells.

### Defective FA and membrane elasticity in Pk1-deficient cells

**Figure 5:**
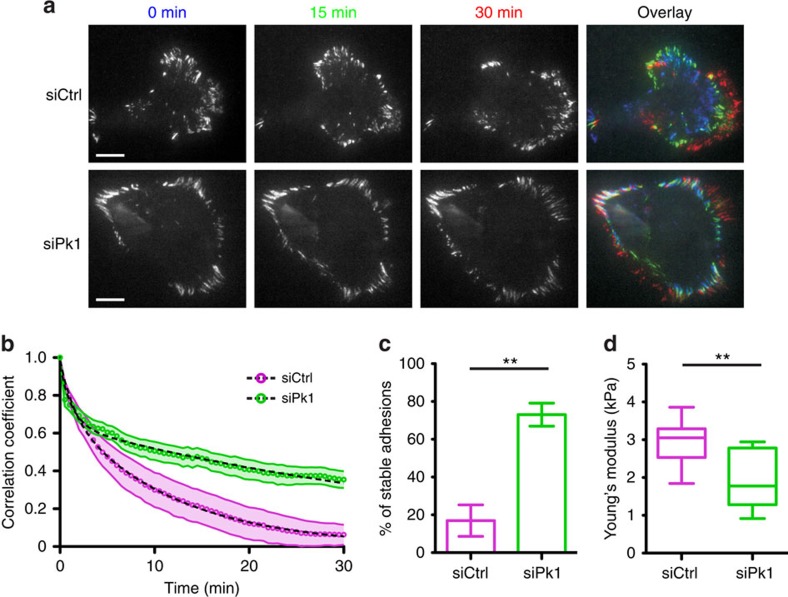
Pk1 regulates FA dynamics and membrane elasticity. (**a**–**c**) Pk1 silencing inhibits FA dynamics. MDA-MB-231 cells were transfected with paxillin-eGFP and the indicated siRNAs. Cells were treated with ACM and the paxillin-eGFP signals were examined by TIRF microscopy to study FA dynamics. (**a**) Representative snapshots of a cell at 0, 15 and 30 min are shown and the right column shows an overlay of the three snapshots pseudo-coloured with blue, green and red, respectively. Note: white colour in the overlay indicates that the signal of the corresponding FA did not change overtime. Scale bar, 10 μm. (**b**) Autocorrelation analysis of time-lapse movies of paxillin-eGFP was performed to examine the resemblance between pixel intensity in the *n*th frame of the movie relative to those in the first frame and the correlation coefficient was plotted over time. Average correlation decays from five movies of control cells and nine movies of Pk1 knockdown cells are shown on the plot. Dashed lines represent double exponential fitting curves and shaded area represents s.e.m. (**c**) The percentage of stable FAs was quantified and results from a representative experiment repeated three times are plotted (*n*=6 cells for siCtrl and 7 cells for siPk1, and results represent mean±s.d. **P<0.001 with two-tailed unpaired *t*-test). (**d**) Pk1-deficient cells display decreased membrane elasticity. MDA-MB-231 cells were transfected with control siRNA or siRNA targeting Pk1. The Young's modulus of cell membrane was measured by AFM as described in Methods. Results of a representative experiment repeated three times are shown (*n*=12 for siCtrl group and 7 for siPk1 group. **P<0.001 with two-tailed unpaired *t*-test).

The aberrant FAs in Pk1- and Arhgaps-deficient cells suggested a defect in their assembly–disassembly cycles. To study FA dynamics, we expressed paxillin-enhanced green fluorescent protein (eGFP) in MDA-MB-231 cells and examined migrating cells using live-cell total internal reflection fluorescence (TIRF) microscopy (Supplementary Movies 5 and 6). In motile control cells, paxillin-eGFP was concentrated in protrusions with few apparent in the lateral cortex, and as protrusions progressed, paxillin-eGFP-marked FAs displayed fast and dynamic changes in both size and morphology that reflected the formation–maturation–turnover cycle (Fig. 5a and Supplementary Movie 5). In contrast, large and stable FAs were found around the entire periphery of Pk1-deficient cells (Fig. 5a and Supplementary Movie 6). This suggests that Pk1 lateral signalling confines RhoA activity to protrusions, which is required for dynamic turnover of FAs. Accordingly, autocorrelation analysis showed remarkably reduced dynamics in paxillin-eGFP signals (Fig. 5b), significantly larger proportions of stable FAs (Fig. 5c) and slower assembly and disassembly rates of FAs (Supplementary Fig. 4a,b) in Pk1-deficient cells. Interplay between the actomyosin network, cell adhesions and the cell membrane is known to regulate biophysical properties that are tightly coupled to the dynamic morphology of migrating cells^25^. The aberrant actomyosin and FA dynamics of Pk1-deficient cells thus further suggested that altered mechanical property might accompany interference with lateral signalling and the acquisition of a static rounded morphology. Consequently, we used atomic force microscopy (AFM) to measure Young's modulus, which reflects the elastic resistance of the cell membrane to deformation forces^26^. This revealed that the loss of Pk1 led to significantly lower Young's modulus compared with the membrane of control cells (Fig. 5d). We conclude that loss of Pk1 leads to decreased FA turnover and loss of cell membrane elasticity.

### Shape volatility correlates with cell migration speed

**Figure 6:**
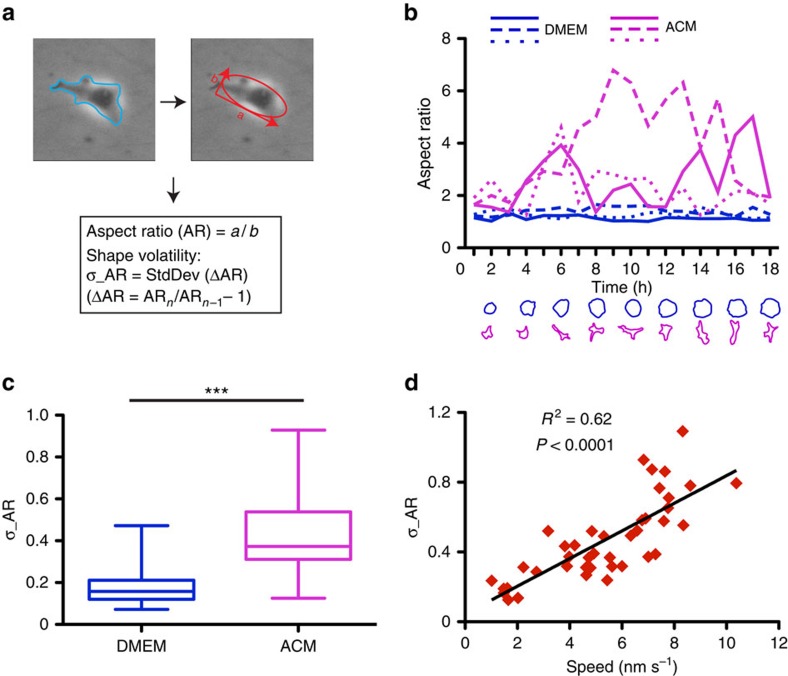
Shape volatility correlates with cell migration. (**a**) Schematic of AR measurement. Snapshots of time-lapse images are used to outline the periphery of motile cells. Individual outlines are fitted into ellipse and AR is calculated as the ratio of the long axis over the short axis. (**b**–**d**) Cell shape volatility correlates with speed during migration. MDA-MB-231 cells were incubated with control DMEM or ACM and imaged for 18 h. For each condition, the AR of three representative cells is plotted over time and the outlines of one representative cell from each condition are shown at the bottom (**b**). The volatility of AR was quantified as described in the Methods and plotted in **c**. Results of one representative experiment repeated three times are shown (*n*=21 cells per group. ***P<0.0001 with two-tailed unpaired *t*-test). The volatility of ACM-treated cells from a representative experiment was plotted against their corresponding migration speed and correlation analysis was performed (**d**).

The mechanical properties of cells are tightly coupled to cell shape^25^,^27^,^28^ and in agreement, we observed that Pk1-deficient MDA-MB-231 cells displayed a stationary morphology that was in stark contrast to the actively changing morphology of ACM-stimulated control cells (Fig. 5a and Supplementary Movies 5 and 6). Furthermore, in comparison with ACM stimulation, cells treated with control media had slower migration and static cell shape (Supplementary Movie 7). Therefore, we next sought to investigate the potential dynamic relationship between cell shape and migration. To quantify morphology, we used the ellipse-fitting algorithm in ImageJ (http://imagej.nih.gov/ij/) to determine the AR of individual migrating cells (Fig. 6a). This revealed a cycling of cell shape between low and high ARs, with individual cells displaying a variety of periodicities and magnitudes of shape changes (Fig. 6b). In contrast, control Dulbecco's modified Eagle's medium (DMEM)-treated cells were static and remained in a low AR state (Fig. 6b). To quantify fluctuations in cell shape, we adapted the concept of financial market volatility to assess the distribution of rates of change in each cell's AR. We define this as shape volatility (method described in the Methods section). Accordingly, ACM-stimulated MDA-MB-231 cells that display greater morphological fluctuations yielded a significantly higher shape volatility index compared with control cells (Fig. 6c). Thus, ACM-mediated activation of migration is accompanied by increased shape volatility. We also confirmed this relationship in ACM-treated human SUM159-PT and T47D cells (Supplementary Fig. 5a,b). Moreover, when we compared shape volatility with speed, we observed strong positive correlation (Fig. 6d). Taken together, these results indicate that shape volatility provides a quantitative measure of cell morphological fluctuations and is correlated with cell migration speed.

### Lateral signalling coordinates shape volatility

**Figure 7:**
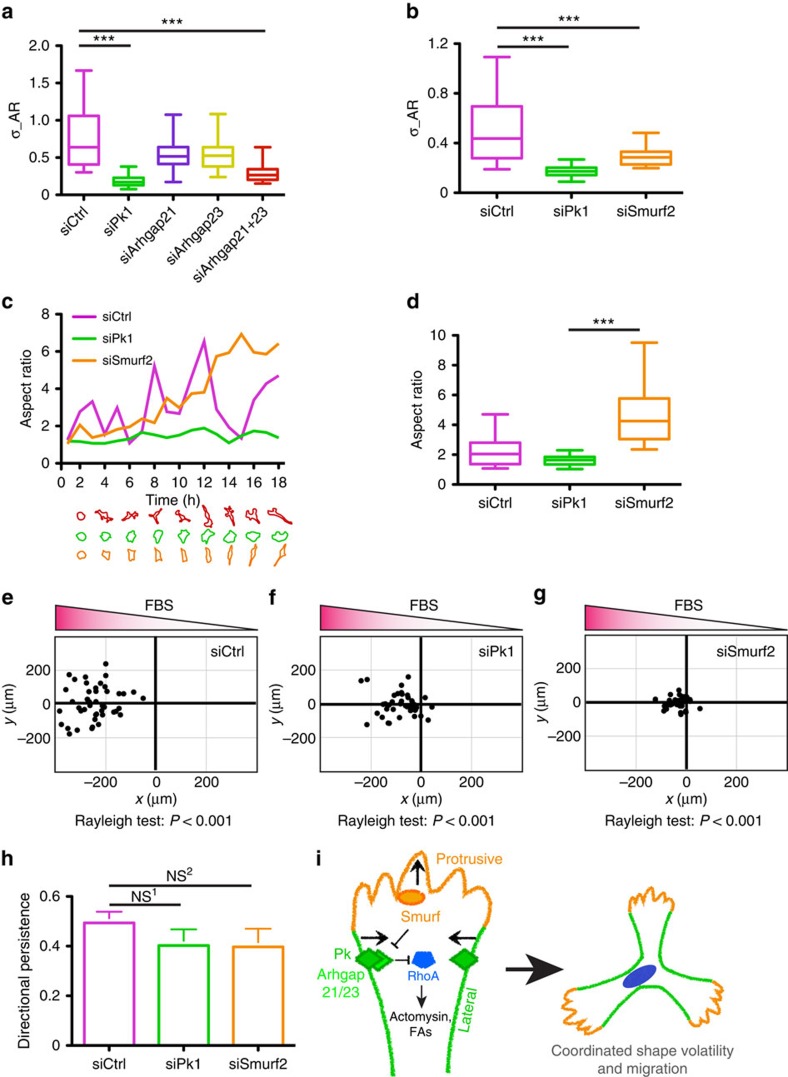
Lateral signalling coordinates shape volatility. (**a**–**d**) MDA-MB-231 cells were transfected with the indicated siRNAs. After 72 h, the cells were treated with ACM for 18 h. The AR volatility of cells in each condition was quantified and plotted in **a** and **b**. A time course of AR of one representative cell in each of the indicated conditions was plotted in **c**, with the cell outlines shown at the bottom. The AR for exosome-stimulated cells at the last time point of the assay was plotted in **d**. Results of one representative experiment repeated three times are shown (*n*=20 cells per group. ***P<0.0001 with two-tailed unpaired *t*-test). (**e**–**h**) Chemotactic migration assays were performed on μ-slide chemotaxis chambers (ibidi) using a FBS gradient between 0.2% (right) and 10% (left). MDA-MB-231 cells transfected with the indicated siRNAs were subjected to directional migration assays and the migration end points of 45 cells are shown in the **e**–**g**. The uniformity of migration endpoints was subjected to the Rayleigh test (**e**–**g**) and the directional persistence of individual cells was analysed by dividing displacement by total distance travelled (**h**). Results from a representative experiment repeated three times are shown (*n*=45 cells per group; NS^1^, *P*=0.095; NS^2^, *P*=0.11 with two-tailed unpaired *t*-test). (**i**) A model depicting how the lateral polarity pathway regulates shape volatility of migrating cells. NS, not significant.

The stationary morphology of Pk1-deficient cells suggests that lateral signalling might regulate shape volatility in migrating cells. Indeed, inhibiting lateral signalling by knockdown of Pk1, Arhgap21 and Arhgap23 led to significantly decreased AR volatility of MDA-MB-231 cells (Fig. 7a). Similarly, enhancing lateral signalling by knockdown of Smurf2 showed similar inhibition of migration and shape volatility (Fig. 7b). However, the two static morphologies were starkly different. While the AR of Pk1 knockdown cells remained low, that of Smurf2-deficient cells progressively increased without major fluctuations (Fig. 7c and Supplementary Movie 8). As a result, while loss of either Smurf2 or Pk1 inhibits shape volatility (Fig. 7b), they do so in different ways, with Smurf2-deficient cells accumulating in a high AR spindle morphology compared with the low AR rounded morphology caused by the loss of Pk1 (Fig. 7d).

Conventionally, asymmetries in migrating cells have been described as front–rear polarity along a solitary axis that connects the leading protrusion and the trailing body^2^. In this context, restricting lateral protrusions and confining the formation of new protrusions at the pre-existing leading front have been proposed as a mechanism that drives directional cell migration^29^. Our results indicate that Pk1 lateral signalling is required to coordinate protrusions and drive shape volatility in random migrating cells with multiple protrusions. We therefore sought to explore the requirement in directional migration using a gradient created between 0.2% and 10% fetal bovine serum (FBS) in μ-slide chemotaxis chambers (ibidi). Under these conditions, control MDA-MB-231 cells undergo efficient chemotaxis towards the higher FBS concentration (Fig. 7e). Interestingly, similar to random, exosome-stimulated migration, knockdown of Pk1 or Smurf2 both significantly inhibited migration speed and, importantly, shape volatility of MDA-MB-231 cells (Fig. 7f,g and Supplementary Fig. 6a,b). However, when we assessed migration direction, both Pk1- and Smurf2-deficient cells maintained oriented migration towards the higher concentration of FBS (Fig. 7f,g, Rayleigh test, *P*<0.001 for all conditions). Furthermore, directional persistence was not significantly different between groups (Fig. 7h). Therefore, while Pk1-mediated lateral signalling is critical for migration speed, it does not have a significant role in the steering mechanism underlying directional migration.

Altogether, our results demonstrate the existence of a novel Pk1 lateral signalling pathway that maintains non-protrusive cortex and confines protrusive activity in cells that undergo both random and directional migration. Lateral signalling dynamically interacts with Smurf-centred signalling and this interplay is required to coordinate shape volatility that drives productive cell migration (Fig. 7i). In this context, when lateral signalling predominates (for example, on Smurf2 knockdown), the cells accumulate a high AR shape with little protrusive activity. In contrast, when lateral signalling is inhibited (for example, loss of Pk1–Arhgap21/23 signalling), the cells acquire a low AR phenotype with concomitant expansion of protrusive activity. In both scenarios, cell migration is inhibited, but importantly this occurs regardless of whether cells are undergoing random versus directed cell migration. These results suggest that the protrusive-lateral signalling network revealed is distinct from signalling pathways that control the direction of migration.

## Discussion

Our studies reveal the existence of a lateral signalling pathway that plays a key role in regulating the dynamics of cell morphology, which we show can be quantified by measuring shape volatility that reflects the distribution of rates of change in the cells' ARs (Fig. 6). We demonstrate that lateral Pk1–Arhgap21/23 signalling interacts with Smurf signalling and serves to coordinate protrusive activities, active RhoA, the contractile actomyosin network, FA dynamics and the mechanical property of cell surface that together drive cell shape volatility (Fig. 7i). Furthermore, we show that shape volatility is coupled to productive cell migration, thus these studies provide insight into how core components of PCP signalling that are typically associated with the planar organization of epithelium can also function to organize the morphological dynamics of single migrating cells.

In PCP signalling, Pk1 engages in a complex with Vangl that antagonizes the Fzd/Dvl complex^30^. In the other direction, a Smurf-Dvl-Par6 complex mediates ubiquitination and degradation of Pk1 (ref. 15). Importantly, Smurf and Par6 are known to promote cell protrusive activities and Dvl co-localizes with Fzd in the multiple protrusions of migrating cells^9^,^16^. Thus, we propose that Pk1-mediated lateral signalling engages in antagonistic interactions with Smurf-Dvl-Par6 protrusive signalling at multiple regions of cell cortex to drive shape volatility. It will be of importance to study how the interactions between protrusive and lateral signalling are temporally and spatially regulated at upstream levels. Furthermore, while severe loss of function strategies in either protrusive or lateral signalling lead to marked extremes of shape changes (Fig. 7c,d), it is intriguing to speculate that modulation of these pathways could drive more moderate shape dynamics that are critical for tissue morphogenesis.

The global output of polarity signalling is the asymmetric organization of cellular structures and behaviour, which depends on the mechanical integration of the biophysical activities manifested by the actomyosin network and cell adhesions regulated by Rho GTPases^8^,^31^. Recently PCP was shown to control the asymmetric compartmentalization of the actomyosin network that directs the convergent extension movements of mesodermal epithelia in Xenopus^32^. Here we provide the first evidence that PCP regulates asymmetry of the actomyosin network and the dynamics of cell adhesions in single, motile cells. Defects in Pk1-mediated lateral signalling led to expansion of protrusive actomyosin networks and stable FAs that displayed slow turnover rates (Figs 4 and 5). These findings suggest a key role of PCP signalling in coordinating the actomyosin network and FAs that integrate with membrane to regulate the mechanical properties of a moving cell.

Rho GTPases play essential roles in cell migration and are regulated by guanine nucleotide exchange factors and GAPs^19^,^20^. Here we show that Pk1 mediates lateral signalling through interacting with RhoGAPs Arhgap21 and 23. The Pk1–Arhgap23 complex constitutively exists inside the cell (Fig. 2e and Supplementary Fig. 2g) and is recruited to the cell cortex on activation of cell migration (Fig. 2f,h). Interestingly, our immunofluorescence results indicate that a substantial population of Arhgap23 does not get recruited to the cell cortex with Pk1, suggesting additional biological functions of this RhoGAP at other cellular compartments.

Cell polarity during migration has been primarily described as a manifestation of front–rear sensing. Indeed, a plethora of signalling networks regulate the asymmetric organization of cellular structures and activities along the front–rear axis of a migrating cell^2^,^4^. In this context, preventing formation of lateral protrusions that extends away from the main front–rear axis is a mechanism that steers directional cell migration^29^. However, in our current study, stimulated cancer cells undergo random migration and rarely displayed a solitary front–rear axis, but rather produced several protrusions in different directions that are bracketed by Pk1 lateral signalling (Fig. 1). Intriguingly, while disruption of lateral signalling leads to marked inhibition of cell shape changes and migration, it does not have a significant effect on the orientation of cell movements in directional migration. This suggests that coordination of shape volatility by lateral signalling orchestrates cell migration orthogonally to conventional front–rear signalling.

Overall, our studies identify a novel lateral signalling system that governs shape volatility and drives productive cell migration. Understanding how lateral signalling and other pathways are integrated to regulate cell morphological plasticity and migration will provide new insights into mechanisms governing tissue morphogenesis in development and their contribution to cancer progression.

## Methods

### Cell culture and ACM preparation

MDA-MB-231 cells were a gift from Dr Robert S. Kerbel (Sunnybrook Health Sciences Centre, Toronto, Canada) and were cultured in RPMI (Gibco) with 5% FBS (HyClone). L cells were purchased from American Type Culture Collection (ATCC; CRL-2648) and were cultured in DMEM (HyClone) with 10% FBS. SUM-159PT cells were purchased from Asterand and were cultured in Ham's F12 medium (Gibco) with 5% FBS, 5 μg ml^−1^ insulin (Sigma-Aldrich), 1 μg ml^−1^ hydrocortisone (Sigma-Aldrich) and 10 mM HEPES. EMT6 cells were purchased from ATCC (CRL-2755) and were cultured in DMEM medium with 10% FBS. T-47D cells were purchased from ATCC (HTB-133) and were cultured in DMEM medium with 10% FBS and 5 μg ml^−1^ insulin. To prepare ACM, confluent L cells were incubated with DMEM supplemented with 50 U ml^−1^ penicillin/streptomycin (Gibco) and 0.25 μg ml^−1^ amphotericin B (Gibco) for 3 days. The conditioned media was then collected, filtered with a 0.2-μm pore size filter (Nalgene) and stored at 4 °C.

### Cell migration assay and shape volatility analysis

For the single-cell migration assay, cells were seeded at low density in assay media prepared from mixing equal volumes of 5% FBS/RPMI with control DMEM or ACM. For the chemotactic migration assay, cells were seeded into chambers on μ-slide Chemotaxis^3D^ (80326, ibidi) with a gradient formed with 0.2 and 10% FBS. Cells were incubated at 37 °C in a humidified chamber supplied with 5% CO_2_ (Pathology Devices, Inc.). Time-lapse movies were collected on a phase-contrast Leica microscope (DMIRE2) with a MS-2000 xyz automated stage (ASI) using a × 10 N-Plan objective lens and an Orca-ER camera (Hamamatsu). Images were captured at a rate of 2 images per hour for 18 h using Volocity software (PerkinElmer). Cell shapes were outlined by using the ‘freehand selection' tool in ImageJ (http://imagej.nih.gov/ij/) to mark the periphery of each cell as region of interest (ROI). Each ROI was then analysed by using the ‘Fit Ellipse' and ‘Measure' function in ImageJ to determine the geometric centre and AR. The speed of a migrating cell was determined by tracing the movement of its geometric centre for 18 h. To calculate the fluctuations of cell shape, we adapted and modified the measure of volatility in finance. Mathematically, shape change at time point *n* is calculated as ΔAR=AR_*n*_/AR_*n*−1_−1, and the shape volatility of a migrating cell is defined as the s.d. of hourly shape changes during the course of experiment: σ_AR=StdDev (data range of ΔAR).

### Immunofluorescence

Cells grown on coverslips were fixed with 4% paraformaldehyde, permeabilized with 0.25% Triton X-100/PBS and blocked with 2% BSA/PBS. The cells were then immunolabelled with primary antibodies (as listed below) followed by fluorescently labelled secondary antibodies (Biotium). Samples were counterstained with 4',6-diamidino-2-phenylindole dihydrochloride (D9542, Sigma-Aldrich) and Alexa-488-phalloidin (A12379, Invitrogen) before mounted onto slides (12-550-143, Thermo Scientific) for visualization. Images were acquired using a × 40/numerical aperture (NA) 1.25 or × 63/NA 1.32 oil immersion objective lens (HCX PL APO, Leica), an EM-CCD camera (ImagEM, Hamamatsu) on an inverted microscope (DMIRE2, Leica) with a spinning disk confocal scanner (CSU10, Yokogawa) and Volocity. Quantifications were performed using ImageJ. The primary antibodies used in immunostaining were as follows: anti-Prickle1 Fab antibody (100 ng ml^−1^)^9^; rabbit anti-cortactin (3503, Cell Signaling, 1:500); rabbit anti-Arhgap23 (HPA019818, Sigma-Aldrich, 1:2,000); rabbit anti-phospho-MLC2 (ab2480, Abcam, 1:100); and mouse anti-paxillin (05-417, Millipore, 1:250). The secondary antibodies used in immunostaining were as follows: CF555-donkey-anti-mouse (20037, Biotium, 1:1,000); and CF647-donkey-anti-rabbit (20047, Biotium, 1:1,000).

### Immunoprecipitation and immunoblotting

Proteins were extracted with TNTE lysis buffer and immunoprecipitation was performed following standard procedures. Total protein lysates and samples from immunoprecipitation were separated by SDS–PAGE, transferred to nitrocellulose membrane (162-0115, BioRad) and probed with primary antibodies (as listed below) followed by horseradish peroxidase-linked secondary antibodies (NA931 and NA934, GE, 1:10,000). The signals were detected using SuperSignal chemiluminescence reagent (34095, Thermo Scientific). The primary antibodies used for immunoblotting were the following: rabbit anti-Arhgap23 (HPA019818, Sigma-Aldrich, 1:2,000); mouse anti-Flag (F3165 Sigma-Aldrich, 1:5,000); rabbit anti-GAPDH (G9545, Sigma-Aldrich, 1:10,000); mouse anti-αTubulin (T6199, Sigma-Aldrich, 1:10,000); mouse anti-T7 (69522, Novagen, 1:10,000); rabbit anti-RhoA (sc-179, Santa Cruz, 1:500); mouse anti-Rac1 (R56220, Transduction Laboratories, 1:2,000); rabbit anti-phospho-MLC2 (ab2480, Abcam, 1:1,000); and mouse anti-MLC2 (M4401, Sigma-Aldrich, 1:1,000). Full blots are available in Supplementary Fig. 7.

### TIRF microscopy

For time-lapse TIRF imaging, MDA-MB-231 cells transfected with paxillin-eGFP and either non-targeting or PK1-targeting siRNAs were cultured on coverslips (Corning) for 4 h in 5% FBS/RPMI with DMEM or ACM. To promote cell attachment and integrin engagement, the coverslips were coated with 10 μg ml^−1^ of human plasma fibronectin (EMD-Millipore) for 2 h at 37 °C and extensively washed with Dulbecco's phosphate buffered saline (DPBS) to remove unbound protein. Before imaging, the medium was changed to CO_2_-independent medium (FBS/RPMI without phenol red supplemented with 10 mM HEPES) and cell sample was mounted on an Eclipse Ti-E inverted microscope (Nikon Instruments) with an evanescent field depth of ∼100 nm. The imaging system was equipped with a CFI Apo TIRF × 60 NA 1.49 objective, a dynamic focusing system (PFS2) to correct for focus drift (PFS2; Nikon Instruments), and a CoolSnap HQ2 CCD video camera (Roper Scientific). All microscope functions were controlled using MetaMorph software (Molecular Devices). During the experiment, the cells were maintained at 37 °C and at 85% humidity with a heated stage-top incubator and an objective heater (Pathology Devices).

Custom scripts written in MATLAB (Mathworks) were used for image analysis to quantify the number of stable and dynamic FAs. Binary masks of individual FAs were created by an automated segmentation of paxillin-eGFP TIRF images using an intensity threshold as described^33^. Briefly, two-dimensional median filter with 10-pixel square window was applied to the time series of the TIRF images to reduce camera noise, and global threshold was computed by using MatLab histogram threshold function. The threshold value was used to convert paxillin-eGFP images to binary segment maps. Next, the resultant binary maps of the FAs were processed by applying MatLab morphological opening function with 3-pixel square kernel followed by filling holes in the segments. Then, the dynamics of the individual segments were followed to identify those that maintained a constant area and paxillin-eGFP fluorescence intensity (the maximal changes did not exceed 10% from the initial value) over the time course of the movie. These segments were considered to be stable FAs. To quantify the fraction of stable FAs in a cell, the number of stable FAs were divided by the total number of FAs.

Autocorrelation analysis of FA dynamics was performed on TIRF movies of paxillin-eGFP as described^34^. In brief, time series of paxillin-eGFP images were converted to binarized masks of FAs as described above, and two-dimensional correlation coefficient was calculated in MatLab by comparing the FAs at each time point with those at first image of the time series. Measurement of FA assembly and disassembly rates was performed as described previously^35^. Briefly, time series of paxillin-eGFP TIRF images were median filtered with a 10-pixel square kernel and total intensity of eGFP fluorescence was measured for each time point by using Metomorph software package (Molecular Devices). The values were plotted on semi-logarithmic scales and the apparent rates of FA assembly and disassembly were determined from the slopes of these graphs.

### AFM and force measurement

All force–distance curves were collected using a Digital Instruments Bioscope scanning probe system and a Digital Instruments Nanoscope IIIA controller with software version 5.30r3sr3. The Bioscope system was mounted onto an Olympus IX-70 inverted microscope base equipped for both confocal and TIRF imaging. ROIs for the force spectroscopy experiments were identified optically using a × 60 objective (PlanApo, Olympus) and the AFM tip centred in the optical field of view. The force curves were acquired using SNL-10 C-tips (nominal spring constant of 0.06 N m^−1^ and nominal tip radius of 2 nm) previously irradiated with ultraviolet light for 15 min. The tips were calibrated for deflection sensitivity on cell-free region of the Willco dishes (64-0759,Warner Instruments). The tip was positioned at the midpoint between the lamellipodium and the nucleus. To prevent cell damage, the tip was raised at least 30 μm above the Willco dish surface before initiating the force spectroscopy experiments. Force spectra were acquired by stepping the tip towards the surface in 1 μm increments and, at each step, collecting a single force curve cycle using a ramp size of 2.5 μm at a tip scan rate of 0.5 Hz corresponding to a tip velocity of 1.25 μm s^−1^. Relative trigger mode was used to avoid possible system drifts due to mechanical causes or thermal effects that increase total applied force. A range of trigger loads from 0.6 to 3.0 nN was considered. All analyses were performed on force curves collected with a trigger force of 1.5 nN. The data collection for each Willco dish took <2 h to ensure the health and mobility of the cells. Young's modulus values were determined after processing the approach curves using Indentation Analysis in Nanoscope Analysis software version 1.40 R3Sr5.96909 with tip half angle of 18.00°, Poisson's ratio of 0.50 and Sneddon model for conical tip.

### Mass spectrometry

Proteins from MDA-MB-231 cells stably expressing Flag-Pk1 were extracted with TNTE lysis buffer. Anti-Flag immunoprecipitation was performed following standard procedures using anti-Flag M2 affinity gel (A2220, Sigma-Aldrich). Samples were reduced with 5 mM dithiothreitol (11583786001, Roche) in 100 mM NH_4_HCO_3_ (40867, Sigma-Aldrich) buffer, pH 8.0, alkylated with 50 mM iodoacetamide (I1149, Sigma-Aldrich) and incubated with trypsin (V5111, Promega) at 37 °C overnight. The tryptic digestion was stopped by adding 5% formic acid and the resulting peptides were analysed by liquid chromatography (Agilent 1100 series) and tandem mass spectrometry using LTQ-XL (Thermo Scientific). Data processing, searching and analysis were performed using ProHits^36^.

### RhoA and Rac1 activation assay

Assays were performed with the RhoA/Rac1/Cdc42 Activation Assay Combo Biochem Kit (Cytoskeleton, Catalogue # BK030) following instructions. Briefly, cells were lysed in the provided lysis buffer 72 h after transfection of siRNAs or 36 h after transfection of plasmids. Cell lysates containing 600 μg total protein were incubated with 25 μg of Rhotekin-RBD or 15 μg of PAK-PBD beads to precipitate active RhoA or Rac1, respectively. The samples were resolved by 12% SDS–PAGE and immunoblotted for RhoA and Rac1. Ponceau red staining was applied to ensure equal sample loading (Supplementary Fig. 7).

### Plasmids, cloning and transfection

Human Pk1 coding sequence (NM_001144881) was PCR amplified in frame with a N-terminal Flag-tag and subcloned into pCAGIP expression vector using MluI and NotI restriction enzymes. The MluI restriction site within the Pk1 coding sequence was destroyed (ACGCGT to GCGCGT) by site-directed mutagenesis into a silent mutation (AGA to AGG). Plasmids encoding 3xFlag-Arhgap23 were kindly provided by Dr Oliver Rocks and Dr Tony Pawson.

MDA-MB-231 cells were transfected with cDNA or shRNA expression vectors using Lipofectamine LTX (Invitrogen) following the manufacturer's instructions. shRNA for human Prickle1 (RHS4430–99616472, Thermo Scientific) was purchased from Open Biosystems. pGIPZ non-silencing shRNA (RHS4346; Thermo Scientific) was used as control shRNA. Cells stably expressing shCtrl, shPk1 or YFP-actin were selected and maintained in culture media containing 2 μg ml^−1^ puromycin.

For siRNA transfection, MDA-MB-231 cells were transfected with 40 nM siGENOME SMARTpool siRNAs (Dharmacon) using DharmaFECT 4 (Dharmacon) transfection reagents following the manufacturers' instructions. Each pool contains four different siRNAs and the pools have always been deconvolved to ensure specificity. Non-Targeting siRNA Pool#2 (D-001206-14; Dharmacon) was used as a control siRNA.

### RNA extraction and real-time quantitative PCR

Total RNA was isolated from cell lysates using RNeasy mini kit (Qiagen) and reverse transcribed with SuperScriptIII reverse transcriptase (18080-044, Invitrogen) using oligo(dT)_18_ primers (Thermo Scientific) following the manufacturers' instructions. Quantitative PCR was performed using SYBR Green I Master Mix (Roche) on a LightCycler 480 II instument (Roche). Primers are listed in Supplementary Table 1. All quantitations were normalized to endogenous GAPDH. Relative changes in gene expression were quantified using the 2^−ΔΔC^_T_ method^37^.

### Statistical analysis

Experiments were repeated at least three times. For quantitative analysis of immunofluorescence data, 10–30 cells were analysed in each repeat. Statistical significance was calculated using two-tailed unpaired *t*-test with the programme Prism (GraphPad Software, Inc.). Values of *P*<0.05 were considered statistically significant. For all box and whisker plots, the line represents the mean, the box represents 25–75 percentile and the whiskers represent min to max.

### Code availability

Custom MatLab scripts used for quantitative image analysis are available from J.L.W. and S.V.P. on request.

### Data availability

The data that support the findings of this study are available from L.Z. and J.L.W. on request.

## Additional information

**How to cite this article:** Zhang, L. *et al*. A lateral signalling pathway coordinates shape volatility during cell migration. *Nat. Commun.* 7:11714 doi: 10.1038/ncomms11714 (2016).

## Supplementary Material

Supplementary Information Supplementary Figures 1-7 and Supplementary Table 1.

Supplementary Movie 1 Migrating cells display lateral polarity. MDA-MB-231 cells stably expressing YFPactin were transfected with control siRNA. Three days later, the cells were treated with ACM and time-lapse image acquisition was initiated at 4 hours after treatment using spinning-disc confocal microscopy (6 sec intervals, 70 frames, 5 fps).

Supplementary Movie 2 Pk1 is required for lateral polarity of migrating cells. MDA-MB-231 cells stably expressing YFP-actin were transfected with siRNA targeting Pk1. Three days later, the cells were treated with ACM and time-lapse image acquisition was initiated at 4 hours after treatment using spinning-disc confocal microscopy (6 sec intervals, 70 frames, 5 fps).

Supplementary Movie 3 Smurf2 silencing leads to expansion of lateral polarity. MDA-MB-231 cells stably expressing YFP-actin were transfected with siRNA targeting Smurf2. Three days later, the cells were treated with ACM and time-lapse image acquisition was initiated at 4 hours after treatment using spinning-disc confocal microscopy (6 sec intervals, 70 frames, 5 fps).

Supplementary Movie 4 Arhgap21 and Arhgap23 are required for lateral polarity of migrating cells. MDAMB-231 cells stably expressing YFP-actin were transfected with siRNAs targeting Arhgap21 and Arhgap23. Three days later, the cells were treated with ACM and time-lapse image acquisition was initiated at 4 hours after treatment using spinning-disc confocal microscopy (6 sec intervals, 70 frames, 5 fps).

Supplementary Movie 5 Dynamics of focal adhesions in migrating cells. MDA-MB-231 cells were transfected with a control siRNA. Two days later, the cells were transfected with a plasmid expressing paxillin-eGFP and were treated with ACM after 24 hours. Time-lapse image acquisition was initiated at 4 hours after cell seeding and treatment using TIRF microscopy (30 sec intervals, 61 frames, 3 fps).

Supplementary Movie 6 Pk1 silencing inhibits focal adhesion dynamics. MDA-MB-231 cells transfected with siRNA targeting Pk1. Two days later, cells were transfected with a plasmid expressing paxillin-eGFP and were treated with ACM after 24 hours. Time-lapse image acquisition was initiated at 4 hours after cell seeding and treatment using TIRF microscopy (30 sec intervals, 61 frames, 3 fps).

Supplementary Movie 7 ACM stimulate shape volatility and cell migration. MDA-MB-231 cells were treated with control DMEM (left panel) or ACM (right panel). Time-lapse image acquisition was initiated at 1 hour after cell seeding and treatment using phase-contrast microscopy (60 min intervals, 18 frames, 6 fps).

Supplementary Movie 8 Silencing of Pk1 and Smurf2 inhibit shape volatility and cell migration. MDA-MB-231 cells were transfected with a control siRNA (left panel) or siRNA targeting Pk1 (middle panel) or Smurf2 (right panel). After 72 hours, cells were treated ACM and time-lapse image acquisition was initiated at 1 hour after cell seeding and treatment using phase-contrast microscopy (60 min intervals, 18 frames, 6 fps).
