# Supplementary material for: A lateral signalling pathway coordinates shape volatility during cell migration
Source: Nat Commun. 2016 May 26;7:11714. doi: 10.1038/ncomms11714 (PMC4894969; doi:10.1038/ncomms11714)
Supplement: Supplementary Information — Supplementary Figures 1-7 and Supplementary Table 1. [file ncomms11714-s1.pdf]

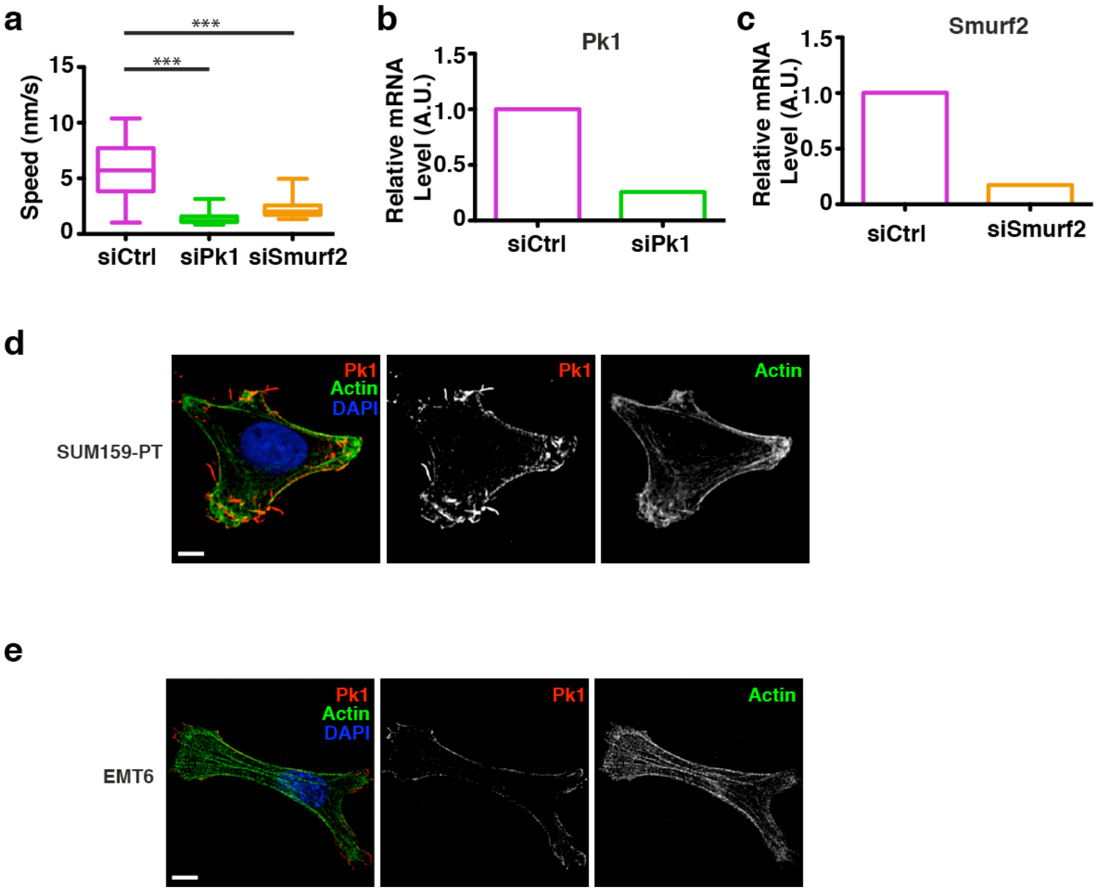

Supplementary Figure 1. (a-c) Pk1 and Smurf2 regulate cell migration. MDA-MB-231 cells transfected with control siRNA or siRNA targeting Pk1 or Smurf2 were treated with ACM for 18 hours. Cell migration speed was quantified and shown in **(a)** (n = 20 cells per group. *** indicates $p < 0.0001$ with two-tailed unpaired t test. Results of one representative experiment from four biological repeats are shown). The mRNA level of Pk1 **(b)** or Smurf2 **(c)** was determined by RT-qPCR and is shown as expression levels relative to cells transfected with control siRNA. **(d-e)** Pk1 localization and lateral polarity in migrating cells. SUM159-PT **(d)** and EMT6 **(e)** cells were treated with ACM and immunostained for Pk1 (red (first column) and white (second column)) and counterstained with phalloidin (green (first column) and white (third column)) and DAPI (blue (first column)). Scale bar = 10 μm.

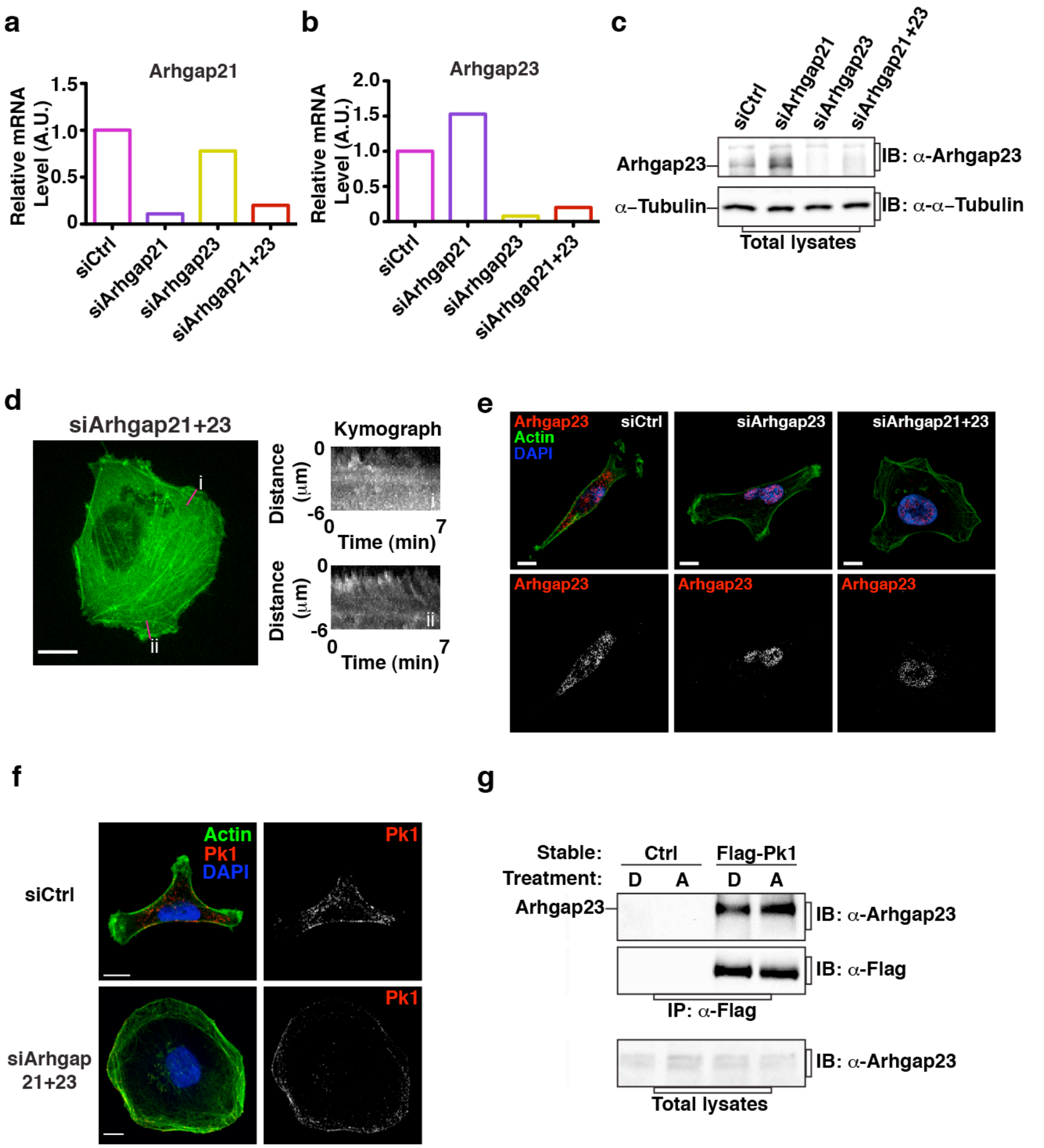

Supplementary Figure 2. Arhgap21 and Arhgap23 regulate lateral polarity. **(a-c)** MDA-MB-231 cells were transfected with control siRNA or the indicated siRNA targeting Arhgap21, and Arhgap23. The mRNA levels of Arhgap21 **(a)**, and Arhgap23 **(b)** were determined by RT-qPCR and are shown as expression levels relative to cells transfected with control siRNA. **(c)** Lysates from MDA-MB-231 cell transfected with the indicated siRNAs were subjected to anti-Arhgap23 immunoblotting (IB). Anti-

αTubulin IB was performed as loading control. **(d)** MDA/YFP-actin cells with combinatorial Arhgap21 and Arhgap23 knockdown were treated with ACM and examined by time-lapse confocal microscopy. Representative snapshots are shown, and kymograph analyses of the regions highlighted by the pink bars are shown in the right panels. White scale bar = 10 μm. **(e)** Analysis of the specificity of the Arhgap23 antibody. MDA-MB-231 cells transfected with indicated siRNAs were immunostained with an Arhgap23 antibody (red (first row) and white (second row)) and counterstained with phalloidin (green (first row)) and DAPI (blue (first row)). Scale bar = 10 μm. **(f)** MDA-MB-231 cells transfected with indicated siRNAs were treated with ACM. Confocal images of immunostained endogenous Pk1 are shown with phalloidin and DAPI staining. Scale bar = 10 μm. **(g)** Lysates from MDA-MB-231 cells stably expressing Flag-Pk1 or control vector (Ctrl) were subjected to the indicated immunoprecipitation (IP) and immunoblotting (IB) assays.

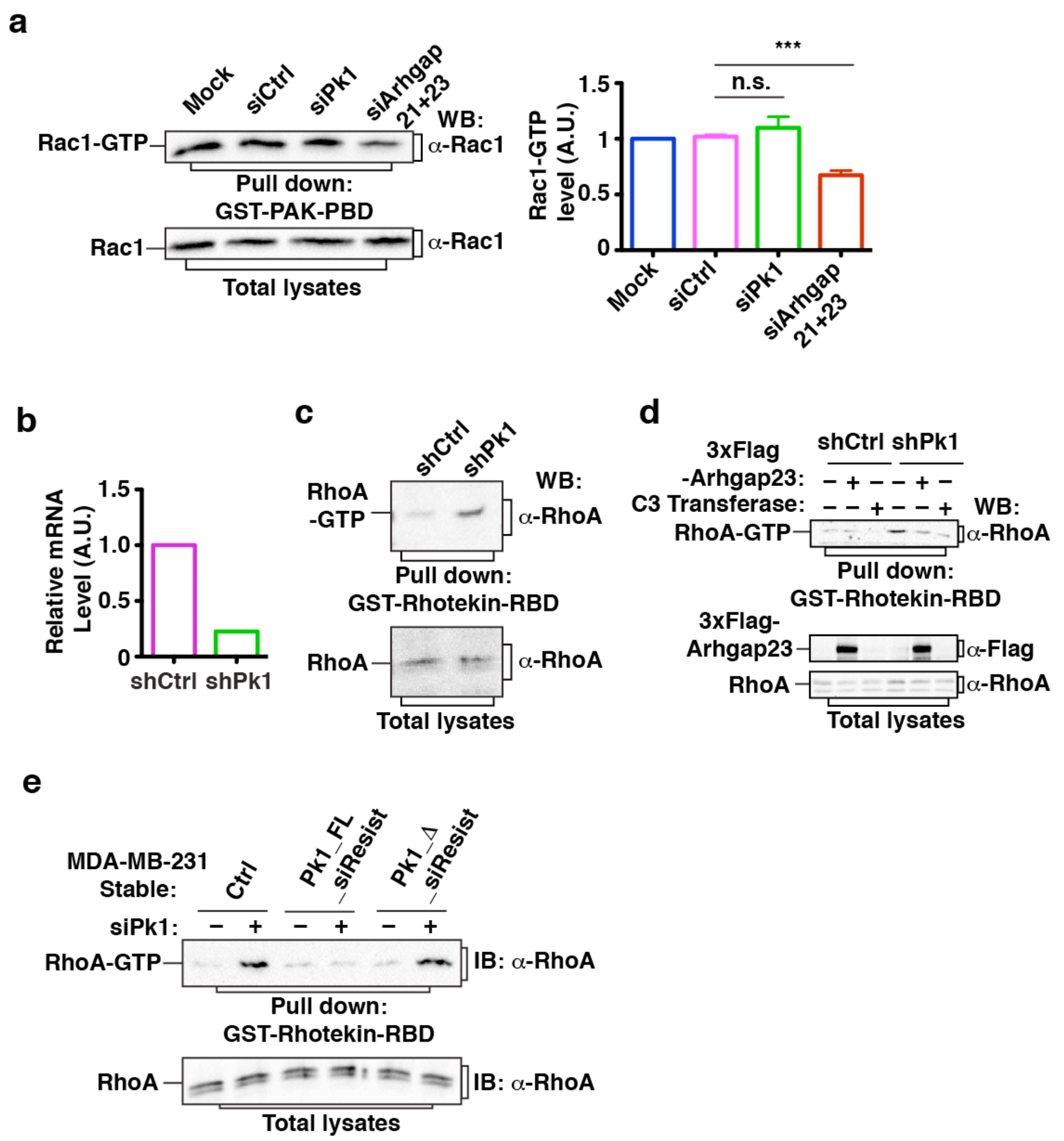

Supplementary Figure 3. Lateral signalling regulates RhoA activity. **(a)** MDA-MB-231 cells were transfected with the indicated siRNAs and the GTP-bound Rac1 in cell lysates was subjected to a pull-down assay using GST-fused PBD (Results represent mean + SD, n = 4 biological repeats, *** indicates $p < 0.001$ and n.s. ($p = 0.45$) indicates not statistically significant with two-tailed unpaired t test). **(b-c)** MDA-MB-231 cells were stably transfected with control shRNA or shRNA targeting Pk1. The expression of Pk1 was evaluated by RT-qPCR **(b)** and the level of active RhoA was examined **(c)**. **(d)**

Arhgap23 decreases the level of active RhoA in Pk1-deficient cells. MDA-MB-231 cells stably expressing control shRNA or shRNA that targets Pk1 were transfected with 3xFlag-tagged Arhgap23 or treated with C3-transferase that inhibits RhoA activity. The level of active RhoA was examined. **(e)** Pk1-Arhgap23 interaction is important for regulating RhoA activity. MDA_FL_siResist, MDA_Δ_siResist, or control (Ctrl) cells were transfected with control siRNA or siRNA targeting Pk1. The level of active RhoA was examined.

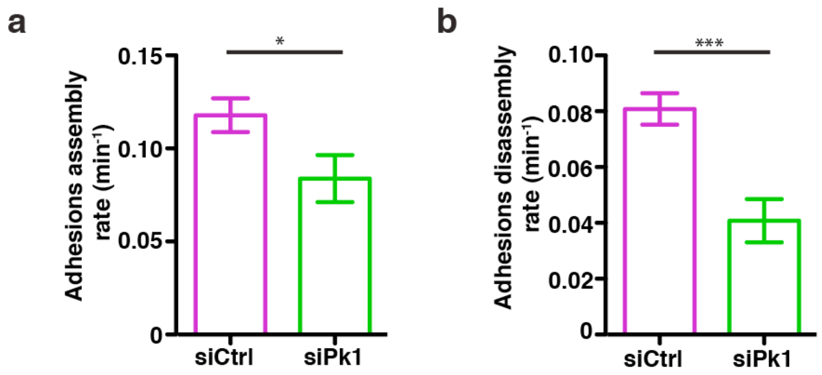

Supplementary Figure 4. Pk1 silencing inhibits the assembly and disassembly rate of focal adhesions. MDA-MB-231 cells from Figure 4a-c were used for measuring the assembly **(a)** and disassembly rate **(b)** of focal adhesions (n = 39 for siCtrl cells, and n = 19 for siPk1 cells. Results represent mean ± SD. * indicates $p < 0.05$ and *** indicates $p < 0.0001$ with two-tailed unpaired t test. Results of one representative experiment from three biological repeats are shown). Note that stable focal adhesions, the ones that did not undergo either assembly or disassembly over the time course of 30 min, were excluded from the analysis.

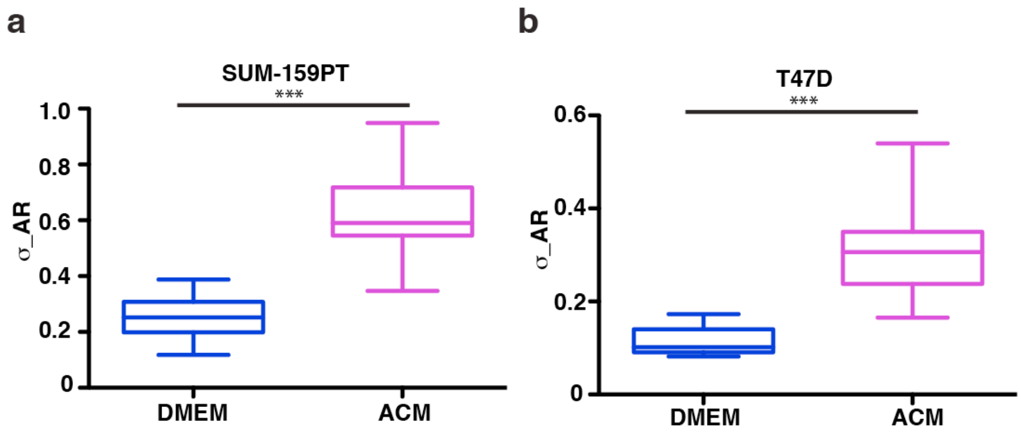

Supplementary Figure 5. ACM stimulates cell shape volatility. SUM159-PT **(a)** and T47D **(b)** cells were incubated with control DMEM or ACM for 18 hours before the volatility of AR was quantified (n = 20 cells per group. *** indicates $p < 0.0001$ with two-tailed unpaired t test. Results of one representative experiment from three biological repeates are shown).

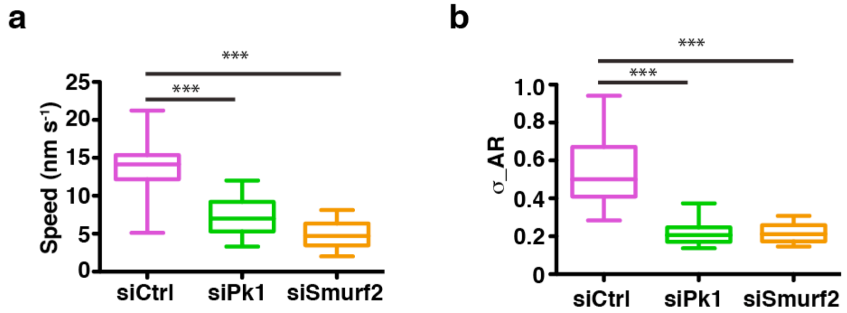

Supplementary Figure 6. Lateral signalling coordinates shape volatility. MDA-MB-231 cells were transfected with the indicated siRNA and subjected to chemotactic migration assays as in Fig. 7e-h. Migration speed **(a)** and volatility of AR **(b)** were quantified (n = 20 cells per group. *** indicates $p < 0.0001$ with two-tailed unpaired t test. Results of one representative experiment from three biological repeats are shown).

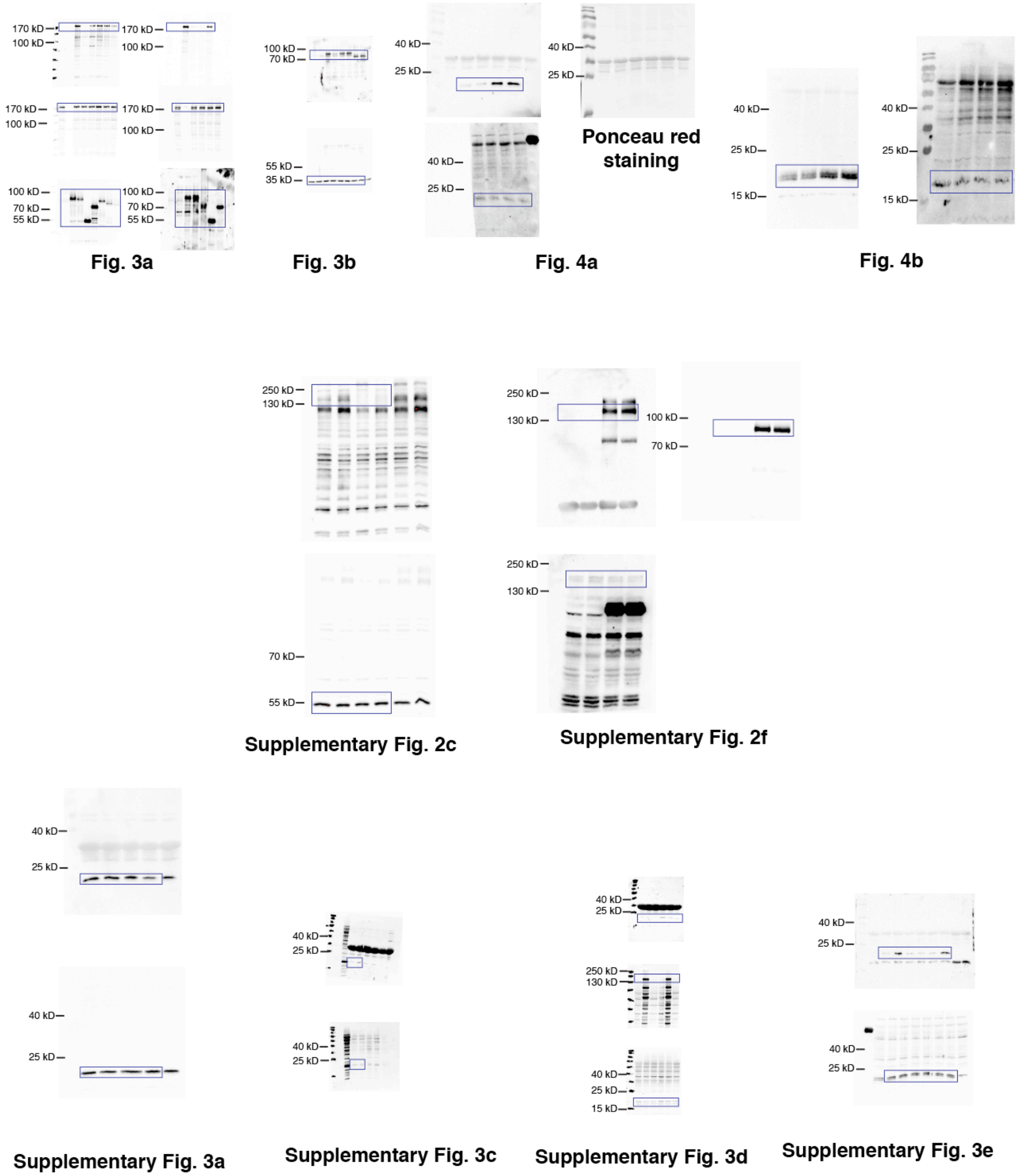

Supplementary Figure 7. Uncropped Western blots related to the indicated figures.

Supplementary Table 1. List of PCR primers.

Gene	Species	RefSeq ID	Primer Sequence 5'-3'	Amplicon Size (bp)
PRICKLE1	Human	NM_001144881	GTCGCCATTGGCACATGAAACACT<br>AGAGGCTTTACACTGGGCACAAGA	239
ARHGAP21	Human	NM_020824	ACAGTGCAGGAGGAAAGCACAGTA<br>ACTAGCAGCTGCAAAGATGGAGGA	207
ARHGAP23	Human	NM_001199417	ACATGACAGACATGGTGACCCACA<br>ACCCTTGGACTTGGCTGAACTACA	239
SMURF2	Human	NM_022739	AAGAGTGCCCAGGGATCTTAGCAA<br>CCACTTGCTGTTGCTGTTGGTCTT	212
GAPDH	Human	NM_002046	AATCCCATCACCATCTTCCA<br>TGGACTCCACGACGTACTCA	82
